# Recent advances in clathrin-independent endocytosis

**DOI:** 10.12688/f1000research.16549.1

**Published:** 2019-01-31

**Authors:** Anupama Hemalatha, Satyajit Mayor

**Affiliations:** 1 Department of Genetics, Yale School of Medicine, New Haven, CT, 06511, USA; 2 National Centre for Biological Sciences (NCBS-TIFR), Bangalore, Karnataka, 560065, India

**Keywords:** Clathrin-independent, endocytosis, actin-dependent, BAR domain

## Abstract

Endocytic pathways are broadly classified into clathrin dependent and independent on the basis of the requirement for the coat protein, clathrin. The molecular pathways and mechanisms underlying the formation of clathrin-independent pathways are still being explored, and this review summarizes recent advances and emerging functional roles of these diverse pathways. In particular, this review will discuss the growing consensus on the role of BAR domain proteins and the actin machinery in different clathrin-independent pathways and its significance to the functions fulfilled by these endocytic pathways.

## Introduction

Endocytosis involves vesicular carriers that bud from the plasma membrane carrying membrane components, including signaling ligands, receptors, nutrients, or growth factors that are delivered to intracellular sites where they are further processed, recycled, or degraded. Secretion and endocytosis are closely integrated with the cellular protein synthesis and degradation machinery such that the flux of material through these processes is a key element in maintaining homeostasis in a cell. Naturally, the diversity in the types of cargo and the kinetics of their trafficking demand a diversity in the types of endocytic machinery employed. The convenient classification based on the requirement for the coat protein, clathrin, divides these endocytic processes into clathrin-dependent and clathrin-independent pathways ^1– 3^. The formation of clathrin-coated pits and associated molecular machinery has been relatively well studied along with its functions in many physiological contexts ^4, 5^. This review will deal with recent findings about the clathrin-independent pathways.

## Molecular machinery underlying clathrin-independent endocytic pathways

### Dynamin-dependent clathrin-independent endocytic pathways

Endocytic pathways that do not use clathrin can either use the scission GTPase dynamin or be independent of it. They are known to be more sensitive to the dynamics of the cytoskeletal actin machinery for stabilization and scission ^2^. Recently, a role for BAR domain proteins has emerged in multiple clathrin-independent endocytosis (CIE) pathways. The members of the BAR domain protein endophilin—primarily endophilin-A2 and endophilin-A1—directly bind to and facilitate the endocytosis of selected G-protein-coupled receptors like β1-adrenergic receptor (β1-AR) and dopaminergic and acetylcholinergic receptors ^6^. Endophilin and lamellipodin have previously been implicated in the clathrin-mediated endocytosis of epidermal growth factor receptor (EGFR) ^7^. During the CIE of β1-AR, rapidly formed vesicles labeled by endophilin and its binding partners, synaptojanin and dynamin, internalized β1-AR from the leading edge of cells in culture and mediated the downregulation of its signaling. This novel endocytic pathway was named the fast endophilin-mediated endocytic pathway (FEME) and requires the lipid phosphatidylinositol 3,4-bisphosphate—PI(3,4)P2—as well as the protein lamellipodin, which binds both PI(3,4)P2 and the SH3 domain of endophilin for its recruitment to the leading edge. The class I PI(3)K pathway and associated phosphatases, including the 5′ phosphatase SHIP1/2, act near the plasma membrane to locally enhance the PI(3,4)P2 levels. This assists in the formation of FEME vesicles enabled by the recruitment of endophilin ^6^.

Endophilin-A2 has also been recently implicated in the CIE of glycosphingolipid (GSL)-binding toxins Shiga (STxB) and cholera toxin B ^8^. These toxins induce local membrane curvature upon binding to their receptor, following which the BAR domain-containing protein is recruited to the cholesterol-dependent curved membrane surfaces ^8, 9^. Cortical actin dynamics were previously shown to be necessary for scission of STxB endosomes ^10^. With reconstituted model membranes, endophilin-A2 was observed to stabilize the tube-shaped StxB vesicles. Actin, endophilin-A2, and dynamin independently and additively contribute to the scission of these vesicles, and the authors propose a model wherein endophilin-A2-stabilized invaginations are acted upon by the pulling forces of cytoskeletal motors (possibly microtubule-associated motors ^11^) to lead to scission at a controlled rate and length ^8^.

### Clathrin- and dynamin-independent endocytic pathways

The reliance on actin cytoskeletal machinery has also been studied in a clathrin- and dynamin-independent, CLIC/GEEC (CG) pathway. This is the major route of internalization of a large fraction of glycosylphosphatidylinositol-anchored proteins (GPI-APs) and fluid phase in several mammalian and *Drosophila* cell lines and tissues ^1, 12– 16^. The CG endocytic intermediates carrying GPI-APs and fluid phase markers were visualized by electron microscopy to reveal uncoated, tubular intermediates ^17^ called clathrin-independent carriers (CLICs), which eventually fuse to form GPI-AP-enriched early endosomal compartments (GEECs) ^18^. Arf1, its GEF (guanine exchange factor), GBF1/Garz ( *gartenzwerg*), and membrane cholesterol are necessary for forming the CG endocytic vesicles in this pathway ^1, 12, 15, 19^. In a screen in S2R ^+^ insect cell lines to identify molecular players regulating this pathway, Arf1-COP1 machinery, BAR domain-containing proteins, vacuolar ATPase, lysosomal genes involved in vacuole biogenesis, and actin remodeling factors, including Slingshot, Coronin, Arcpc1, and Capping protein, were among the major hits ^19^. Actin inhibitors used at concentrations that disrupt the cortical actin network without affecting the long-lived structures like stress fibers selectively perturb CG endocytosis while not affecting clathrin-dependent uptake ^12^. This acute sensitivity to actin dynamics also translates to the regulation of CG endocytosis by Cdc42, a modulator of actin polymerization.

Recently, the recruitment of these early molecular players at nascent endocytic vesicles was studied at high spatial and temporal resolution to understand the sequence of events leading to the formation of a CG endocytic vesicle ^20^. While reaffirming the role of ARF1-GBF1, the actin nucleator Arp2/3, and associated GTPase Cdc42 at the nascent sites of endocytosis, this study also identifies the role of a BAR domain protein, IRSp53, and an ARP2/3 inhibitor, PICK1. Imaging the direct recruitment of these molecular players also helps develop a mechanistic picture of how a GEEC is formed: initial recruitment of ARF1/GBF1, IRSp53, and Arp2/3 to the cell surface is followed by the arrival of Cdc42 that activates Arp2/3 and IRSp53 to catalyze the formation of F-actin and membrane buckling, leading to the formation of the CG endosome ^20^. The ARF1/GBF1 ( *Garz*)-dependent CG pathway was also shown to be active in *Drosophila* wing disc tissue, wherein class I PI3-kinase activity is necessary for the recruitment of *Garz* to the cell surface and the initiation of the CG endocytosis ^16^.

Besides the role of the actin machinery, membrane composition is a key component that determines the enrichment of clathrin-independent cargoes in endocytic pits ^2^. Perturbations in cholesterol and sphingolipid levels affect the distribution of GPI-APs, normally present as actively maintained nanoclusters on the cell surface ^21^, and subsequently the formation of GEEC endosomes ^12^. The CIE of cholera and Shiga toxins too is initiated upon their binding to specific GSLs, which induces membrane bending and endosome formation ^8, 9, 22^. Endogenous galectins, which can bind glycan chains and GSLs, are postulated to use a similar mechanism for concentration and multimerization leading to membrane bending, potentially facilitating the internalization of a large variety of endogenous glycosylated proteins. This GSL-galectin-3-mediated mechanism (termed GL-Lect ^22^) can mediate the CIE of β1-integrin and CD44, dependent on their glycan chains ^23^. Recently, galectin-3 binding was also implicated in the endocytosis of the GPI-AP CD59 and major histocompatibility complex (MHC) class I protein, suggesting a more global utilization of this axis of interaction to lend specificity to CIE ^24^. Interestingly, the degree of glycan branching affects the internalization rates in contrasting ways. CD59 internalization is obstructed by a highly branched glycan lattice. This raises the possibility of clathrin-independent endocytic fluxes being selectively altered by factors affecting N-glycosylation, like metabolic substrates ^24^.

## Functional roles of clathrin-independent endocytosis

Emerging understanding of the molecular machinery behind non-canonical endocytic processes provides a view of the versatility of CIEs depending on the context. Clathrin-independent rapid endocytosis (~100 ms) at the synapses of *Caenorhabditis elegans* neurons ^25^ and also in mouse hippocampal neurons ^26^ is necessary for compensating and coordinating membrane retrieval with exocytosis rates ^27^. This ultra-fast endocytosis is triggered by exocytosis, and recently synaptojanin-1 and endophilin-A have been implicated in the rapid maturation of these endocytic pits so as to facilitate membrane turnover in milliseconds—an essential feature of synapses ^28^.

Plasma membrane cholesterol is necessary for the functioning of many CIEs, including CG and Shiga toxin endocytosis. Cholesterol-dependent CLICs were recently found to be enriched at the mid-body during cytokinesis at the intercellular bridge by electron microscopy. This postulates a role for CLICs to stabilize and maintain the membrane reservoir during cell division ^29^.

Down-regulation of signaling by CIE has also been documented for EGFR and other ubiquitinated cargoes: at low ligand concentration, clathrin-mediated uptake is employed; at higher ligand concentration, the CIE pathway operated to down-regulate signaling ^30^. A recent role for promoting Wingless/Wnt signaling in developing *Drosophila* wing discs highlights the potential role of CIEs during development and cell fate determination in metazoans ^16^. The previously described CG pathway internalizes and brings together the apically localized ligand Wingless with its basolaterally localized receptor Dfrizzled2 (internalized by clathrin-mediated uptake) to activate full-strength signaling. This novel mechanism of cooperating endocytic pathways that promote signaling could be functional in other contexts where the dosage of signaling needs to be graded rather than act as an on/off switch. The requirement for class I PI3-kinase activity for CG endocytosis and hence Wingless signaling illustrates how this endocytic pathway introduces new nodes of control and modulation from the single modality of ligand–receptor interaction in a tissue ^16^. The well-documented roles of GPI-APs (cargo for CG endocytosis), Dally, and Dlp as potential co-receptors for multiple signaling molecules during development ^31– 33^ also make this pathway an exciting candidate as an integrator of signaling with cues from the actin cytoskeletal machinery and membrane composition in the growing organ.

An actin-dependent macro-pinocytic pathway is upregulated in cancer cells in a nutrient-sensitive manner. Specifically, in Ras-driven tumors, growth factor signaling appears to activate this mechanism. This helps in nutrient scavenging from surrounding extracellular material and even necrotic cell debris to supplement increased energy demand ^34– 36^. This pathway shares some similarities with the CG pathway in its cargo: the fluid phase and its molecular players, actin and PI(3,4,5)P3. However, it is also able to accommodate large dextran (>70 kDa), is amiloride sensitive, and appears specifically upregulated by Ras-mediated activation of Rac and Cdc42, triggered in some cases by AMPK (5′-AMP-activated protein kinase) ^36^.

CLICs are reported to internalize a large fraction of the total plasma membrane surface area; their endocytic volume is capable of internalizing the entire surface area of the plasma membrane in 12 minutes in fibroblasts ^37^. The extreme reliance of CIEs on the actin cytoskeletal machinery makes them a good candidate to transduce information from the mechanical tension experienced by the cell to its homeostatic machinery. A recent study has explored the dependence of the flux in the CG pathway of endocytosis on mechanical tension and finds a reciprocal relationship between the two ^38^. The CG pathway is specifically upregulated transiently to buffer a decrease in mechanical tension during stimulated stretch-relax cycles as well as during normal de-adhering of cells. Conversely, inhibiting CG endocytosis reduced the membrane tension within the cell. This newly uncovered function of the CG endocytic pathway makes it a crucial cog in the homeostatic machinery that maintains cellular mechanical tension and, by extension, its shape, size, and response to the environment. Depleting a molecular player implicated in CIE-Graf1 leads to increased cancer cell blebbing and enhances its invasiveness in culture ^39^, which could be linked to the function of CIEs in maintaining membrane tension and also highlights the many ramifications this process could have in multiple tissue contexts if perturbed. The extent to which the functions of these pathways influence signaling networks and their interdependence remains to be explored, and recent work indicates that there is significant cross-talk ^28^.

Thus, the common emerging theme among CIEs is their reliance on the cell cytoskeletal machinery, especially the cortical actin network, for the early steps. This dependence also provides these endocytic pathways with the ability to respond to rapid changes in the cellular environment. Perhaps the cortical actin network provides the feedback for the control of these endocytic pathways, integrating the endocytic machinery with the dynamic cortical actin mesh.

## Author information

Anupama Hemalatha was affiliated with the National Center for Biological Sciences, Bangalore, India through the conceptualisation and writing of this article, however she is currently affiliated with the Yale School of Medicine, CT, USA.
